# The APOE4-estrogen-microglia axis in perimenopausal cognitive changes: mechanisms and therapeutic implications

**DOI:** 10.3389/fimmu.2026.1925850

**Published:** 2026-08-20

**Authors:** Liuqing Shi, Chuntong Zhou, Huan Han, Mei Lu, Mingjie Li, Junjing Miao, Shiyu Feng, Xin Zhou, Haiyang Chen, Lu Ren

**Affiliations:** 1 Department of Acupuncture and Moxibustion, Liaoning University of Traditional Chinese Medicine, Shenyang, China; 2 Liaoning University of Traditional Chinese Medicine, Shenyang, China

**Keywords:** Alzheimer’s disease, ApoE4, cognitive decline, estrogen deficiency, microglia, neuroinflammation, perimenopause

## Abstract

Alzheimer’s disease (AD) exhibits marked sex differences, with women bearing a disproportionate burden of disease, particularly after midlife. Among the major factors contributing to this vulnerability, the apolipoprotein E ϵ4 allele (APOE4) and the abrupt endocrine transition of perimenopause have emerged as two critical and potentially synergistic drivers of neurodegeneration. Microglia, the resident immune cells of the central nervous system, lie at the center of this interaction because they integrate genetic, hormonal, metabolic, and inflammatory signals that shape amyloid clearance, synaptic remodeling, and neuroimmune homeostasis. Accumulating evidence indicates that APOE4 impairs microglial phagocytosis, disrupts lipid handling and lysosomal function, promotes pro-inflammatory activation, and compromises neurovascular integrity. In parallel, estrogen normally restrains microglial inflammatory signaling and supports phagocytic, metabolic, and reparative functions through estrogen receptor-dependent pathways. During perimenopause, fluctuating estrogen deficiency removes these protective constraints, thereby increasing the susceptibility of microglia to APOE4-driven dysfunction. This review synthesizes current evidence supporting an integrated pathogenic framework centered on the “APOE4-estrogen deficiency-microglia axis.” We discuss how this axis promotes chronic neuroinflammation, synaptotoxicity, amyloid and tau pathology, mitochondrial dysfunction, blood–brain barrier disruption, and large-scale network disconnection, ultimately accelerating cognitive decline in women. We also summarize relevant experimental models, including APOE-targeted murine paradigms, ovariectomy and accelerated ovarian failure models, human induced pluripotent stem cell-derived microglia, and emerging single-cell and spatial omics approaches. Finally, we highlight translational opportunities, including precision hormone-based interventions, selective estrogen receptor modulation, TREM2-centered microglial therapies, APOE4-directed molecular and genetic strategies, and biomarker-guided multi-target interventions during the perimenopausal “window of opportunity.” By integrating molecular mechanisms with translational perspectives, this review proposes a precision medicine framework for preventing or delaying neurodegenerative progression in high-risk perimenopausal women.

## 1 Introduction

Alzheimer’s disease (AD) is the leading cause of dementia worldwide and is characterized by progressive cognitive decline accompanied by amyloid-beta (Aβ) deposition, tau pathology, synaptic dysfunction, and chronic neuroinflammation (1, 2). Although age is the most important risk factor, biological sex significantly influences disease susceptibility, progression, and clinical presentation. Women not only account for a greater proportion of AD cases (3, 4), but also undergo gradual depletion of oocytes and progressive decline in ovarian function during the perimenopausal transition, culminating in the cessation of ovarian activity. Unlike the persistently low estrogen levels observed after menopause, perimenopause is characterized more prominently by pronounced hormonal fluctuations; this unstable endocrine state constitutes an important physiological basis and is associated with a higher risk of disease onset (5, 6). These systemic alterations may have profound effects on the brain, particularly on regions involved in memory and cognition (7). In animal studies, the 5×FAD transgenic mouse model is widely used; it carries five familial mutations across two AD risk genes and exhibits increased Aβ production and plaque formation (8). Notably, sex differences have also been observed in 5×FAD mice, with female mice showing earlier increases in inflammatory gene expression and glial markers (9, 10).

The ϵ4 allele of the apolipoprotein E gene (APOE4) is the strongest and most consistently replicated genetic risk factor for AD (11). APOE3 is the neutral isoform and is not associated with increased risk, whereas approximately 10–15% of the general population carries at least one APOE4 allele. Carrying one ϵ4 allele (E3/E4) may increase the risk of AD by two- to threefold, whereas carrying two ϵ4 alleles (E4/E4) may raise the risk by as much as 10- to 15-fold (12). Importantly, APOE4 also acts as an upstream driver of multiple pathogenic cascades, linking amyloid pathology, tau pathology, microglial dysfunction, lipid dysregulation, neuroinflammation, and vascular impairment (13–17). Despite its central pathogenic role, no targeted therapeutic intervention for APOE4 is currently available. At the same time, estrogen exerts neuroprotective effects through its receptors and downstream signaling pathways, including the regulation of APOE expression and the suppression of neuroinflammation. However, the APOE4 genotype alters the effects of estrogen signaling, rendering carriers, especially women, more vulnerable to estrogen deficiency (18).

Estrogen deficiency may provide a “second-hit” condition for APOE4-mediated pathogenic mechanisms. Microglia occupy a central position in this interaction. Early microglial responses were initially considered protective, as they can restrain plaque growth and limit amyloid-associated neuronal injury (19). However, APOE4 has been shown to accelerate neurodegeneration by altering microglial responses (20). Studies have shown that the 151-amino-acid N-terminal fragment of ApoE4 (nApoE4_1-151_) localizes to the nuclei of microglia in human AD brain tissue, where it induces cytotoxicity and promotes microglial activation, and has been shown in cell-based experiments to upregulate the expression of inflammatory genes such as IL-12B and CXCL2 (21). Accordingly, the association between the APOE4 allele and dementia risk may be mediated, at least in part, by microglial activation that exacerbates inflammation. In female-derived activated response microglia (ARMs), a distinct subpopulation characterized by the expression of genes involved in MHC class II antigen presentation, tissue repair, and AD genetic risk (including APOE and BIN1) has been associated with progressive Aβ accumulation (22). In recent years, single-cell transcriptomic studies have revealed marked microglial heterogeneity and have identified a microglial subpopulation closely associated with female sex and AD pathology, termed female-associated disease-associated microglia (FDAMic) (23). We propose that the combined effects of APOE4-related microglial dysfunction and the perimenopausal decline in estrogen levels constitute a pathophysiological axis that may help explain the heightened neuroinflammatory responses and increased vulnerability to cognitive impairment observed in women during midlife.

In this review, we integrate evidence from molecular, cellular, animal, and clinical studies to examine the “APOE4-estrogen deficiency-microglial axis” in perimenopausal neuroinflammation and cognitive decline. We further discuss emerging experimental models, therapeutic potential, and the translational challenges that must be addressed to advance precision medicine for women at elevated risk.

## 2 Perimenopause: a window of neuroimmune vulnerability

Women exhibit marked sex differences in AD risk, onset, progression, and severity (24). Perimenopause, the transitional phase characterized by irregular ovarian hormone secretion and menstrual cyclicity preceding the onset of menopause, may represent a particularly vulnerable period for the development of mild cognitive impairment and AD (25–27). Perimenopause constitutes a distinct state of inflammatory “priming,” in which hormonal fluctuations interact synergistically with inflammation, lipid accumulation, and vascular alterations (28). During this period, the loss of estrogen’s anti-inflammatory effects may exacerbate the activation of nuclear factor kappa B (NF-κB)-related pathways and increase the sensitivity of the NOD-like receptor family pyrin domain containing 3 (NLRP3) inflammasome (29). The principal hormone secreted by the ovaries is 17β-estradiol (E2), which serves as a master regulator of the female brain because of its broad effects on neural structure and function (30). Estradiol receptors, primarily estrogen receptor alpha (ERα) and estrogen ERβ, are distributed in brain regions involved in reproductive and cognitive functions and are also thought to participate in immune regulation (31). Both ERα and ERβ are expressed in microglia and astrocytes, where they are involved in neuroinflammatory processes and are associated with AD (32). Hormonal fluctuations may disrupt estrogen receptor-related functions and, by affecting neuronal cholesterol homeostasis and the tricarboxylic acid cycle, may increase glutamate release, ATP depletion, and susceptibility to energetic crisis (33), thereby further elevating AD risk. Perimenopause should therefore be regarded as a critical biological window during which endocrine alterations intersect with neuroimmune vulnerability and AD risk.

## 3 Roles of estrogen signaling pathways in microglial homeostasis

### 3.1 Estrogen receptors and anti-inflammatory signaling pathways in microglia

The decline in estrogen levels during perimenopause increases women’s susceptibility to AD, a process closely linked to microglial dysfunction caused by attenuated estrogen signaling (34). Estrogen exerts broad neuroprotective effects through multiple receptor systems, including estrogen ERα, ERβ and membrane-associated receptors such as GPER (35). As the resident immune cells of the brain, microglia play a central role in AD pathogenesis; they cluster around Aβ plaques and exhibit transcriptomic features associated with neurodegeneration (36). Activation of estrogen receptor beta has been shown to suppress microglial activation and attenuate neuroinflammation in amyloid-depositing mouse models, with this protective effect exhibiting sex specificity (37). In animal models, estrogen and related compounds act through microglial ERα and ERβ to regulate microglial inflammatory states and metabolic activity, thereby delaying AD pathological progression (38). In recent years, single-cell transcriptomic studies have revealed heterogeneous microglial states in AD, among which the discovery of disease-associated microglia (DAM) has been particularly important; the progressive transition from homeostatic microglia to DAM depends on triggering receptor expressed on myeloid cells 2(TREM2) (39). In addition, a functionally impaired, FDAMic is abnormally expanded in female patients with AD, and its transcriptomic profile indicates disrupted estrogen receptor signaling, underscoring the importance of estrogen receptor signaling in microglial functional regulation (23). Estrogen can suppress NF-κB activation, inhibit NLRP3 inflammasome signaling, regulate the MAPK and PI3K/Akt pathways, and promote transcriptional programs associated with repair and homeostatic maintenance (40). In contrast, estrogen deficiency may lower the activation threshold for NF-κB and NLRP3 inflammasome pathways, impair the expression or function of receptors involved in phagocytic adaptation, and compromise mitochondrial function, collectively driving microglia from a homeostatic surveillance state toward a more reactive state with reduced reparative capacity (34). By suppressing excessive inflammatory signaling and supporting adaptive responses, estrogen helps maintain microglial function under stress.

### 3.2 Regulation of phagocytosis, polarization, and immunometabolism by estrogen

In the early stages of AD, microglia exert protective effects by clearing Aβ and tau through phagocytosis; however, as the disease progresses, persistent inflammatory and oxidative stress conditions can lead to excessive microglial activation, thereby triggering neuroinflammation, oxidative stress, and neurotoxicity, which further exacerbate neurodegeneration (41). In women carrying the APOEϵ4 allele, attenuation of estrogen signaling may further worsen deficits in lipid metabolism, phagocytosis, autophagy, and bioenergetic adaptation, thereby amplifying the neuroinflammatory cascade (42). Microglia-specific overexpression of transcription factor EB (TFEB) has been shown to restore autophagy-lysosomal pathway (ALP) function, enhance phagolysosomal clearance of oligomeric Aβ, reduce amyloid burden in the cortex, hippocampus, and entorhinal cortex of 5×FAD mice, and improve memory deficits (43). In addition, p62 suppresses NLRP3 inflammasome-mediated proinflammatory responses and pyroptosis by recognizing and binding ubiquitinated NLRP3 and delivering it to the ALP for degradation, thereby preserving microglial phagocytic capacity (44). In AD, microglial IL-33 signaling induces, through epigenetic and transcriptomic reprogramming, a microglial subpopulation with enhanced phagocytic activity, termed IL-33-responsive microglia (IL-33RMs), a process that depends on the binding of the transcription factor PU.1 to target gene DNA (45). Moreover, TREM2, an AD risk gene specific to myeloid cells, is essential for microglial function; its loss reduces the survival of human microglia, impairs the phagocytosis of key substrates including APOE, and inhibits SDF-1α/CXCR4-mediated chemotaxis, ultimately weakening the response to Aβ plaques (46). The MS4A family proteins are a class of transmembrane proteins (e.g., MS4A4A and MS4A6A), predominantly expressed in microglia, and have been shown to cooperatively suppress TREM2 signaling, thereby attenuating protective microglial responses (47). By contrast, the protective MS4A6A allele is associated with increased gene expression, and MS4A6D deficiency leads to a marked reduction in microglial amyloid encapsulation and phagocytosis (48). Because phagocytosis, migration, and lysosomal processing are all highly energy-demanding functional processes, the metabolic support provided by estrogen is crucial for maintaining microglial functional integrity.

## 4 APOE4 as a driver of microglial dysfunction

### 4.1 Dysregulated lipid metabolism in microglia

In the context of AD, dysregulated lipid metabolism in microglia is closely associated with the APOE4 genotype. Disruption of lipid processing contributes to chronic inflammation, impaired Aβ clearance, mitochondrial dysfunction, and synaptic deficits (49). Domain interaction between the N-terminal and C-terminal regions of ApoE4 promotes a pathological folding state, thereby impairing lipid transport and disrupting membrane homeostasis in brain cells (50). These biochemical properties can compromise lipid transport and membrane homeostasis in brain cells, including microglia. Microglia expressing APOE4 exhibit altered cholesterol handling, lipid droplet accumulation, enhanced triglyceride metabolism, and impaired intracellular lipid homeostasis, all of which are closely linked to inflammatory priming, phagocytic deficits, and defective degradation of engulfed material (51, 52). APOE4 may also activate calcium-dependent phospholipase A2 and disrupt arachidonic acid metabolism, thereby increasing the production of proinflammatory eicosanoids and sustaining a state of chronic neuroinflammation (51).

### 4.2 Impaired microglial phagocytosis and defective Aβ clearance

As the resident immune cells of the central nervous system, microglia play a critical role in maintaining brain homeostasis through phagocytosis, including the clearance of Aβ aggregates, apoptotic cellular debris, and dysfunctional synaptic components (53). In transgenic mouse models carrying APOE4, microglial Aβ clearance is impaired, as reflected by reduced uptake of both soluble and insoluble Aβ plaques (54). This defect intensifies the neurotoxic cycle: diminished Aβ clearance directly promotes plaque formation, whereas inefficient removal of apoptotic and damaged cellular material prolongs local inflammatory signaling (55). In addition, TREM2 is an immune receptor predominantly expressed on microglia, and disruption of TREM2-dependent responses shifts microglia from a protective response state toward a dysfunctional proinflammatory phenotype, thereby further impairing their ability to clear Aβ. By interfering with the function of the TREM2-ApoE signaling axis, the APOE4 genotype may exacerbate these effects (56, 57).

### 4.3 Proinflammatory polarization and the antigen-presenting phenotype

In the early stages of AD, microglia can adopt a relatively protective response state, characterized by enhanced Aβ uptake and clearance, as well as support for functions related to tissue homeostasis and repair. As the disease progresses, particularly in the APOE4 background, microglia undergo marked metabolic and transcriptional reprogramming, shifting from a homeostatic maintenance program primarily driven by oxidative phosphorylation to an inflammation-associated activated state that relies more heavily on glycolysis, accompanied by sustained increases in inflammatory mediators such as IL-1β and TNF-α, thereby exacerbating neuronal injury (58–60). The transition of microglia from a homeostatic state to a disease-associated, inflammation-amplifying phenotype is considered one of the major driving forces of chronic neuroinflammation in AD (61). In APOE4-associated tauopathy mouse models, microglia display increased cholesterol biosynthesis and accumulation, which is closely associated with sustained elevation of MHC II-dependent antigen presentation and subsequent increases in T-cell infiltration into the brain (62). Transcriptomic analyses have shown that nApoE4_1–151_ treatment significantly enriches inflammatory pathways, including Toll-like receptor signaling, chemokine/cytokine signaling, and apoptosis-related signaling (21). nApoE4_1-151_ can also directly drive the expression of the key inflammatory cytokine tumor necrosis factor alpha (TNF-α) by binding to its promoter region, increasing its expression by as much as 68-fold and thereby triggering morphological activation and functional transformation of microglia (63). In addition, this fragment influences the neuroinflammatory microenvironment by regulating the DNA methylation status of core genes in chemokine signaling pathways, such as AKT1 and MAP2K1 (64).

## 5 The APOE4-estrogen deficiency -microglia axis: an integrated pathogenic framework

**Figure 1:**
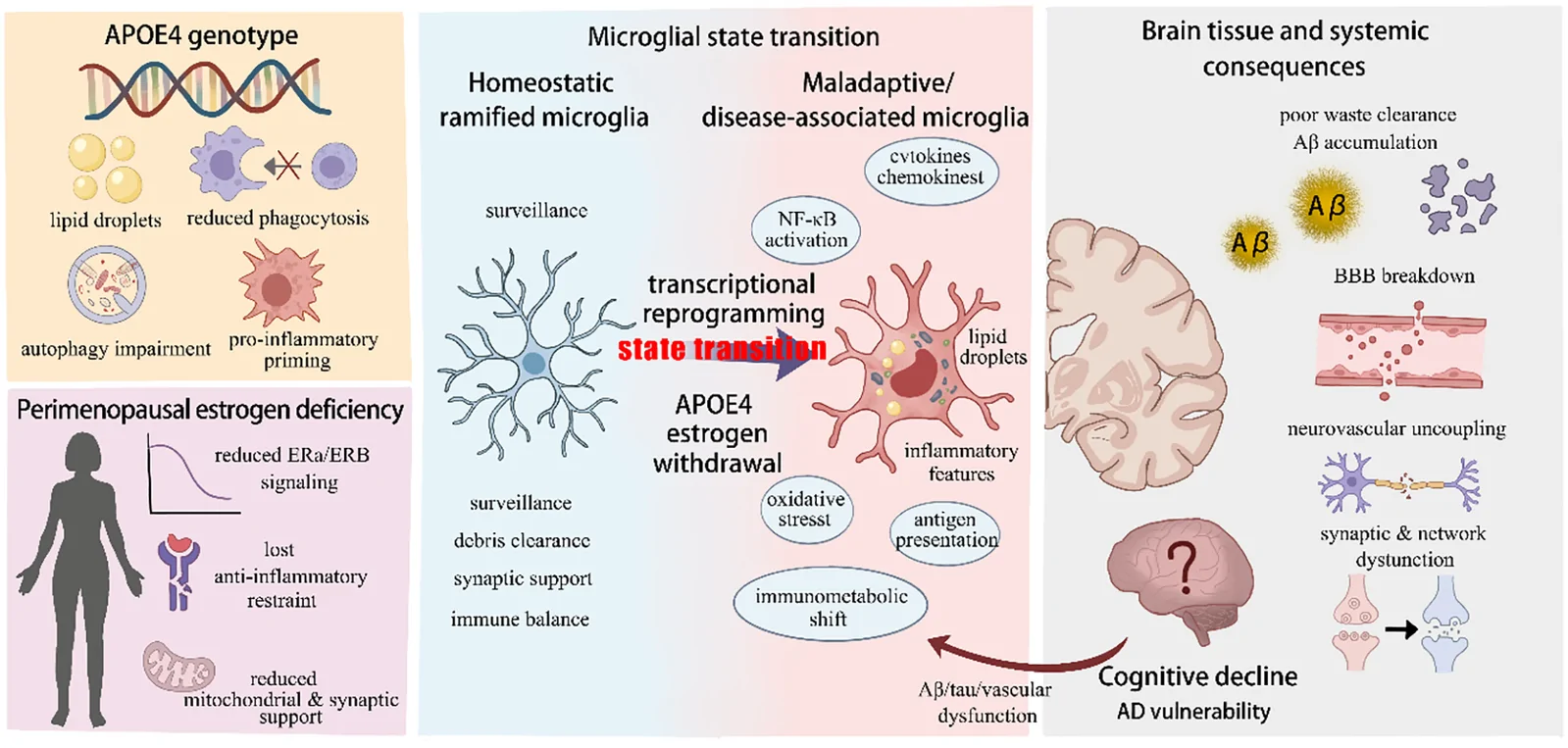
Schematic overview of the APOE4-estrogen-microglia axis in perimenopausal cognitive vulnerability. APOE4 genotype and perimenopausal estrogen deficiency converge to reprogram microglial states from homeostatic surveillance toward maladaptive, disease-associated, and antigen-presenting phenotypes. Reduced estrogen receptor (ER)-mediated restraint, together with APOE4-related defects in lipid handling, phagocytosis, and autophagy, promotes inflammatory amplification and impaired waste clearance. These changes contribute to amyloid-β accumulation, blood–brain barrier breakdown, neurovascular uncoupling, synaptic dysfunction, and brain network instability, ultimately increasing chronic neuroimmune imbalance and cognitive vulnerability.

APOE4 first confers an initial vulnerability upon microglia, rendering their lipid metabolic homeostasis fragile and lowering the threshold for inflammatory responses (65). The loss of estrogen further removes a critical anti-inflammatory brake, making microglia more likely to cross the boundary of adaptation and enter a proinflammatory and dysfunctional state when confronted with Aβ stimulation (66). A central downstream event triggered by activation of this axis is the formation of a self-reinforcing vicious cycle between neuroinflammation and synaptotoxicity. IL-1β, TNF-α, and reactive oxygen species released by activated microglia can directly disrupt synaptic structure and function, whereas damaged synapses expose complement components such as C1q and C3 and release “eat-me” signals, thereby recruiting and further activating additional microglia and establishing a positive feedback loop (67, 68). This aberrant synaptic pruning is particularly prominent in the hippocampus and prefrontal cortex, directly compromising the neuronal network functions required for memory consolidation and working memory (69) (**Figure 1**).

The APOE4-estrogen deficiency-microglia axis also accelerates the canonical pathological progression of Aβ and tau through multiple mechanisms. Chronic inflammation can promote amyloidogenic processing by enhancing β-secretase (BACE1) activity and altering amyloid precursor protein (APP) cleavage, while the APOE4 protein itself is less efficient in binding and clearing Aβ, thereby further contributing to extracellular Aβ accumulation (70, 71). At the same time, inflammatory cytokines can activate kinases such as CDK5 and GSK3β, directly promoting the hyperphosphorylation of tau at multiple pathogenic sites, including Ser396 and Ser404 (72). In addition, APOE4 and the elevated follicle-stimulating hormone (FSH) levels observed during perimenopause may synergistically activate the C/EBPβ/δ-secretase signaling pathway while simultaneously enhancing the proteolytic processing of APP and tau, thereby promoting Aβ generation and neurofibrillary tangle formation (73). Complement-mediated synapse elimination represents a critical link between inflammation and neural circuit degeneration: the combined effects of estrogen deficiency and APOE4 promote the tagging of vulnerable synapses by complement proteins and drive dysregulated microglial pruning, which may help explain why early cognitive symptoms emerge before substantial neuronal loss becomes evident (74, 75).

However, dysfunction of this integrated axis extends far beyond inflammation within the brain parenchyma. Both the APOE4 genotype and estrogen deprivation adversely affect endothelial function, cerebral blood flow regulation, and the integrity of the blood-brain barrier (BBB), thereby impairing nutrient delivery and waste clearance, including Aβ efflux, and increasing the brain’s exposure to peripheral inflammatory signals (76). At the systems level, these processes lead to large-scale disruption of functional connectivity: APOE4 carriers may exhibit altered brain connectivity and increased regional vulnerability even in the absence of substantial Aβ burden, whereas declining estrogen levels further aggravate this network breakdown by weakening vascular and metabolic support (77).

This integrated framework has major implications for understanding female susceptibility to AD. Such susceptibility arises from the complex interplay among sex hormone transition, APOE4-associated genetic risk, and maladaptive microglial responses. In women carrying APOE4, the perimenopausal rise in follicle-stimulating hormone (FSH) and the sharp decline in estrogen resulting from ovarian functional decline may trigger the proinflammatory transformation of microglia decades before the onset of clinical symptoms, thereby initiating the pathological cascade of AD (78, 79). Accordingly, APOE4-positive women constitute a distinct high-risk subgroup, and the critical window of perimenopause is emerging as a primary target for preventive intervention. A summary of the relevant findings is provided in **Table 1**.

**Table 1:** Convergent mechanisms linking APOE4 and perimenopausal estrogen deficiency tomicroglial dysfunction and cognitive decline.

Mechanistic domain	APOE4-related effect	Estrogen deficiency-related effect	Microglial consequence	Downstream brain outcome	Reference(s)
Lipid metabolism	Impaired cholesterol transport, lipid droplet accumulation, altered APOE lipidation	Reduced metabolic support and altered lipid regulatory signaling	Lipid-stressed, dysfunctional microglial states	Chronic neuroinflammation, impaired debris handling	(51, 80, 81)
Phagocytosis and lysosomal function	Reduced Aβ uptake, defective lysosomal degradation, impaired TREM2-associated adaptation	Loss of estrogen-supported phagocytic efficiency	Decreased clearance of Aβ, apoptotic debris, and damaged synapses	Amyloid accumulation, prolonged inflammatory signaling	(82–84)
Inflammatory signaling	Increased pro-inflammatory transcriptional programming, enhanced cytokine production	Reduced suppression of NF-κB and inflammasome pathways	Sustained inflammatory activation with poor resolution	Synaptic injury, neuronal stress, cognitive dysfunction	(34, 59, 85, 86)
Mitochondrial bioenergetics	Mitochondrial dysfunction, oxidative stress, reduced metabolic flexibility	Decline in estrogen-mediated mitochondrial protection and ATP support	Energetically compromised microglia with impaired surveillance and repair	Oxidative damage, impaired resilience, accelerated brain aging	(87–90)
Synaptic regulation	Increased propensity for maladaptive activation and complement-associated pruning	Loss of estrogenic support for synaptic plasticity and anti-inflammatory balance	Excessive or mistargeted microglial synapse elimination	Synaptic dysfunction, memory impairment	(75, 91, 92)
Amyloid pathology	Reduced Aβ clearance and altered APOE–Aβ interactions	Greater inflammatory milieu favoring amyloidogenic processing	Inefficient plaque containment and persistent activation around plaques	Increased amyloid burden and local neurotoxicity	(59, 84, 93–95)
Tau-related pathology	Promotion of inflammatory kinase signaling and tau vulnerability	Hormonal changes may enhance tau-related stress signaling	Cytokine-rich environment favoring tau phosphorylation and spread	Neurofibrillary pathology and neurodegeneration	(96–98)
Neurovascular integrity	Blood–brain barrier impairment, endothelial dysfunction, reduced clearance pathways	Reduced vascular protection and impaired cerebral perfusion	Amplified inflammatory exposure and poor waste clearance	BBB breakdown, neurovascular uncoupling, network dysfunction	(99–103)
Transcriptional state transitions	Bias toward disease-associated and antigen-presenting phenotypes	Reduced ER-mediated restraint on inflammatory gene expression	Shift from homeostatic to maladaptive microglial states	Chronic neuroimmune imbalance and cognitive vulnerability	(104, 105)

Aβ, amyloid-β; BBB, blood–brain barrier; ER, estrogen receptor; NF-κB, nuclear factor kappa B; TREM2, triggering receptor expressed on myeloid cells2.

## 6 Experimental models and emerging technologies

As summarized in **Table 2**, the experimental models and emerging technologies currently used to investigate the APOE4-estrogen deficiency-microglia axis mainly include rodent models, human iPSC-derived systems, single-cell and spatial omics technologies, as well as gene editing and advanced delivery platforms. Each of these approaches offers distinct advantages and can be used to elucidate the underlying mechanisms from different perspectives, including the organismal level, the cellular level, transcriptional heterogeneity, and interventional strategies.

**Table 2:** Experimental models and enabling technologies for studying the APOE4–estrogen deficiency–microglia axis.

Model/technology	Main features	Major strengths	Key limitations	Typical applications	Reference(s)
APOE-targeted replacement mice	Human APOE isoforms introduced into murine background	Controlled genotype-specific mechanistic analysis	Species differences in microglial biology and menopause physiology	APOE isoform effects on inflammation, cognition, and pathology	(106–108)
EFAD or other amyloid-transgenic APOE models	APOE genotype combined with amyloid pathology	Allows study of genotype–amyloid interactions	May overemphasize amyloid-driven pathology	Amyloid deposition, plaque-associated microglia, therapeutic testing	(109–112)
Ovariectomy models	Surgical estrogen depletion	Simple and reproducible induction of estrogen loss	Does not fully mimic fluctuating human perimenopause	Estrogen deprivation effects on neuroinflammation and cognition	(113, 114)
Accelerated ovarian failure models	Transitional ovarian dysfunction closer to menopause-like state	Better approximation of endocrine transition than ovariectomy alone	Still imperfect representation of human perimenopause	Hormone fluctuation studies and timing-of-intervention experiments	(115)
iPSC-derived microglia	Human microglial-like cells generated *in vitro*	Human genetic context, scalable, editable	Incomplete maturation and simplified environment	APOE genotype studies, phagocytosis, inflammatory assays	(116–118)
Isogenic APOE-edited iPSC lines	APOE3/APOE4 comparison in matched background	Strong causal inference for genotype effects	Requires sophisticated engineering and validation	Direct testing of APOE-dependent phenotypes and rescue strategies	(119, 120)
Neuron–astrocyte–microglia co-culture	Multicellular interaction systems	Captures cell–cell crosstalk better than monocultures	Still lacks full tissue architecture	Synaptic pruning, inflammatory communication, hormone response	(121–123)
Brain organoids/assembloids	3D human tissue-like models	Improved structural and multicellular complexity	Variability, incomplete vascularization, developmental immaturity	Network effects, multicellular pathology, therapeutic screening	(124, 125)
scRNA-seq/snRNA-seq	Single-cell transcriptomic resolution	Identifies microglial subpopulations and state transitions	Snapshot data, computational complexity	Disease-associated states, sex-biased signatures, target discovery	(126–129)
Spatial transcriptomics	Spatially resolved gene expression	Links cell states to plaques, vessels, and circuit	Lower resolution than some single-cell approaches	Mapping microglial heterogeneity in tissue context	(130–133)
Lipidomics/metabolomics	Quantification of lipid and metabolic pathways	Highly informative for APOE- and estrogen-related biology	Requires specialized platforms and interpretation	Immunometabolism, lipid droplet states, biomarker discovery	(134–136)
CRISPR screening/gene editing	Functional interrogation of causal genes	Powerful for target validation and pathway mapping	Delivery, off-target concerns, translational barriers	APOE correction, ER signaling studies, pathway dissection	(137, 138)
BBB-on-chip/neurovascular models	Engineered microphysiological systems	Useful for studying vascular and barrier interactions	Simplified relative to *in vivo* human brain	BBB permeability, clearance pathways, neurovascular inflammation	(139, 140)

BBB, blood–brain barrier; ER, estrogen receptor.

## 7 Therapeutic potential of the APOE4-estrogen-microglia axis

**Table 3** summarizes the currently applied and emerging therapeutic strategies targeting the APOE4-estrogen-microglia axis. These interventions include precision hormone therapy, ERβ-targeted compounds, microglia-directed immunomodulation, APOE4-specific molecular and genetic interventions, combination therapies, lifestyle modification, and biomarker-guided risk stratification. Together, these approaches constitute a precision medicine framework aimed at identifying high-risk women during the perimenopausal window and implementing timely multimodal interventions to delay or prevent the progression of neurodegenerative pathology.

**Table 3:** Therapeutic strategies targeting the APOE4-estrogen-microglia axis in perimenopausal cognitive vulnerability.

Therapeutic category	Representative strategies	Putative mechanism of action	Potential benefits	Key caveats/considerations	Reference(s)
Hormone-based intervention	Timed HRT, transdermal estradiol, individualized regimens	Restores estrogenic anti-inflammatory, mitochondrial, and synaptic support	May reduce neuroimmune vulnerability if given within a critical window	Response likely depends on timing, formulation, and APOE genotype	(34, 141, 142)
Selective estrogen signaling	SERMs, ERβ-selective agonists, GPER-directed approaches	Preserves beneficial estrogen receptor signaling with potentially fewer systemic risks	More targeted modulation of neuroinflammation and cognition	Clinical efficacy in APOE4 carriers remains insufficiently defined	(37, 143)
Microglia-targeted therapy	TREM2 agonists, inflammasome inhibitors, PI3K/Akt modulators, immune-rebalancing approaches	Enhances phagocytosis, reduces maladaptive activation, promotes reparative states	Directly targets the central convergence hub of pathogenesis	Risk of oversimplifying microglial biology or causing unintended immune effects	(144–151)
APOE4-directed therapy	Structural correctors, APOE mimetic peptides, lipidation enhancers, antisense/gene-editing approaches	Reduces APOE4 toxicity or corrects APOE4-specific dysfunction	May address a core upstream driver of female AD vulnerability	Delivery, long-term safety, and translational feasibility remain challenges	(93, 96, 152–154)
Proteostasis and autophagy support	Autophagy enhancers, lysosomal modulators	Improves degradation of Aβ, damaged organelles, and inflammatory substrates	May restore microglial clearance competence	Requires careful dosing and disease-stage-specific evaluation	(43, 155, 156)
Metabolic intervention	Ketogenic support, mitochondrial enhancers, insulin-sensitizing strategies	Compensates for impaired brain energetics and supports immunometabolic resilience	May improve midlife brain energy balance and inflammatory control	Adherence and patient selection are critical	(157–162)
Vascular protection	Blood pressure control, lipid management, endothelial support strategies	Improves neurovascular function and BBB stability	Reduces vascular amplification of neurodegeneration	Often under-integrated in mechanistic neurodegeneration studies	(163–166)
Lifestyle-based intervention	Exercise, sleep optimization, Mediterranean-style diet, omega-3 fatty acids, stress reduction	Modulates inflammation, vascular health, metabolism, and synaptic plasticity	Safe and broadly applicable adjunctive support	Effect sizes may vary and require sustained implementation	(167–171)

ER, estrogen receptor; GPER, G protein-coupled estrogen receptor; HRT, hormone replacement therapy; TREM2, triggering receptor expressed on myeloid cells2; BBB, blood–brain barrier.

## 8 Conclusion

The combined effects of the APOE4 genotype and perimenopausal estrogen deficiency provide a biologically plausible and increasingly well-supported framework for understanding female susceptibility to neuroinflammation and cognitive decline. Microglia occupy a central position within this framework, integrating lipid, inflammatory, metabolic, and hormonal signals to influence synaptic integrity, amyloid and tau pathology, neurovascular stability, and brain network function. APOE4 impairs microglial phagocytic capacity, lipid homeostasis, autophagic function, and inflammatory regulation, whereas estrogen deficiency removes critical anti-inflammatory effects and bioenergetic support. Together, these factors drive a persistent shift of microglia toward a maladaptive state, thereby potentially accelerating the progression of neurodegeneration.

Viewing the perimenopausal transition as a “window of opportunity” carries important medical implications. It suggests that timely, stratified interventions, rather than delayed and generalized treatment, may be required to modify disease trajectories in high-risk women. Precision hormone therapy, microglia-targeted therapeutic approaches, APOE4-directed interventions, and biomarker-guided combination strategies all show considerable promise. To fully realize this potential, the field must address the gap between animal and human models, refine female-specific disease models, and conduct carefully designed stratified clinical studies. Precision medicine strategies centered on the APOE4-estrogen deficiency-microglia axis may ultimately improve the prevention and treatment of perimenopausal women at high risk for AD.
